# Conceptualisation and Development of a Quality of Life Measure for Parents of Children with Autism Spectrum Disorder

**DOI:** 10.1155/2014/160783

**Published:** 2014-03-20

**Authors:** Valsamma Eapen, Rudi Črnčec, Amelia Walter, Kwok Ping Tay

**Affiliations:** ^1^Academic Unit of Child Psychiatry, South Western Sydney Local Health District (AUCS), Liverpool Hospital, Mental Health Centre (Level 1: ICAMHS), Locked Bag 7103, Liverpool BC, NSW 1871, Australia; ^2^University of New South Wales, Sydney, NSW 2052, Australia

## Abstract

Parents of children with autism spectrum disorder (ASD) tend to experience greater psychological distress than parents of typically developing children or children with other disabilities. Quality of Life (QoL) is increasingly recognised as a critical outcome measure for planning and treatment purposes in ASD. There is a need for ASD-specific QoL measures as generic measures may not capture all relevant aspects of living with ASD. This paper describes the conceptualisation and development of an autism-specific measure of QoL, the Quality of Life in Autism Questionnaire (QoLA) for parents and caregivers of children with ASD, that is suitable to clinical and research settings. Preliminary psychometric properties (reliability and validity) of the measure are also presented. The QoLA has 48 items in two subscales: one comprising QoL items and the second a parent report of how problematic their child's ASD symptoms are. A study involving 39 families suggested the QoLA has excellent internal consistency as well as good known-groups validity between parents of children with ASD and those who were typically developing. The QoLA also showed good convergent validity with other measures of QoL and ASD symptom severity, respectively. The QoLA may be a valuable assessment tool and merits further psychometric evaluation.

## 1. Introduction

Autism spectrum disorder (ASD) is a lifelong neurodevelopmental disorder characterised by impairments in social interaction, verbal and nonverbal communication, and a restricted repertoire of activities and interests [1]. The prevalence of ASD appears to be rising worldwide [2] with ASD estimated to affect around 1 in every 88 persons [3]. Building on genetic vulnerability [4], it has been hypothesised that ASD emerges from a developmental cascade in which a deficit in attention to social stimuli leads to impaired interactions with primary caregivers. This results in abnormal development of the neurocircuitry responsible for social cognition, which in turn adversely affects later behavioural and functional domains dependent on these early processes [5, 6].

Parenting children with ASD can be emotionally, socially, financially, and physically challenging. Studies have consistently found higher levels of psychological distress in parents of children with ASD when compared to parents of typically developing children or children with other disabilities [7–10]. For example, mothers of individuals with ASD have been shown to display lower levels of wellbeing and higher levels of stress than mothers of individuals with Down syndrome, fragile X syndrome, and cerebral palsy [7, 8]. Similarly, families of children with ASD are reported to experience higher levels of family stress and more family problems than families of children with Down syndrome [11, 12], attention deficit hyperactivity disorder [13], and medical conditions such as cystic fibrosis [14].

In addition to measuring mental health related factors such as stress and psychological wellbeing, a broader and encompassing construct that is important to assess in families of children with ASD is Quality of Life (QoL). In 1995 the World Health Organisation defined QoL as an individual's “perception of their position in life in the context of the culture and value systems in which they live and in relation to their goals, expectations, standards and concerns” [15]. The term “Quality of Life” first appeared in the literature in around 1920, but it was not until the middle of the century that the concept became a part of public discourse. This increased awareness of QoL followed the duel effects of the World Health Organisation broadening the definition of “health” within its 1948 constitution to include physical, emotional, and social wellbeing [16], and of a generally increased cognisance of social inequity in Western societies [17]. Wood-Dauphine notes that between 1966 and 1974 there were 40 references related to QoL identified within a MEDLINE database search, whereas between 1986 and 1994 there were over 10,000—highlighting the proliferation of research, including in the development of generic QoL measures, that began during the 1970s [17, 18]. A wide range of disorder-specific QoL measures have been developed in recent years given the general acknowledgement of the importance of tailoring QoL measures to specific populations [19].

QoL is a multidimensional construct and includes health- and non-health-related domains of functioning. Various models of QoL are available with differing foci, including some that concentrate on health-related aspects of QoL; however, a widely accepted model for assessing QoL in the context of disabilities has been put forward by Schalock et al. [20]. This model proposes eight core QoL domains: personal development, self-determination, interpersonal relations, social inclusion, rights, emotional wellbeing, physical wellbeing, and material wellbeing.

QoL thus offers the potential to be a useful index of parents' adjustment to their child's ASD and is an important component in obtaining a full understanding of the experiences of, and difficulties faced by, individuals with ASD and their families [21–23]. Research in this area is also important as QoL has been identified as a potential mediator of psychological adjustment and treatment outcome for people with ASD and is also pertinent to service planning and evaluation [21].

While advances in the accurate diagnosis of ASD at increasingly earlier ages have been made, as well as the development of evidence-based early interventions [24, 25], QoL research in ASD is in its infancy. In this regard, a recent review of the literature on QoL measures for use in ASD identified a lack of focus on QoL in ASD research and suggested that a new condition-specific measure of QoL was needed [26].

Recent studies have used generic QoL measures to assess QoL among parents whose children have ASD, including the World Health Organisation QoL measures [27]; Short-Form Health Surveys [28, 29]; and EuroQol Five Dimensional Questionnaire (EQ-5D-3L, [30]). Khanna et al. found that the EQ-5D-3L showed good convergent, discriminant, and known-groups validity, and satisfactory internal consistency, when used among caregivers of children with ASD [30]. Similarly, when parents are asked to make QoL ratings on behalf of a young person, generic measures tend to be used [31]. Several instruments, such as the Beach Centre Family Quality of Life Scale [32], have been developed for families of children with intellectual and developmental disabilities and may have some relevance and utility to the ASD population. One ASD-specific QoL measure that has been developed for parents of children with ASD is the 102-item Quality of Life Scale [33], although this measure is presently only available in French.

While generic QoL measures often have large normative data sets and well-articulated psychometric properties underpinning their use, such measures may not prove sufficiently sensitive to the unique challenges faced by individuals with ASD or parents of children with ASD, particularly with respect to social and emotional aspects [34]. Therefore, the power of studies investigating treatment effects may be affected, and the ability to enhance knowledge where QoL is a dependent variable may be compromised. Similarly, within clinical contexts the ability to use a QoL measure to help target and assess treatment outcomes is reduced when a generic measure is utilised. For example, in the context of ASD, generic QoL measures or measures developed for use in intellectual disability do not typically ask specific questions regarding the impact of, say, restricted/repetitive behaviours; sensory difficulties; or difficulties with social understanding.

The present study aimed to develop an autism-specific measure of QoL, the Quality of Life in Autism Questionnaire (QoLA) for parents of children with ASD that is suitable to clinical and research settings. The clinical utility of such a measure would be improved by having a relatively small number of items, as well as subscales that are relevant to service and treatment planning: these were goals of the proposed measure. Furthermore, the present study aimed to investigate the preliminary psychometric properties of the QoLA. The internal reliability of the measure (the consistency of scores across items) was assessed, in addition to its known-groups validity (an index of construct validity measuring a questionnaire's ability to discriminate between groups); concurrent validity (the correlation of the QoLA with an existing validated measure of QoL); and convergent validity (the extent to which scores on the QoLA were associated with scores on theoretically-related measures).

## 2. Materials and Methods

### 2.1. Ethical Approval

The study had the approval of the Human Research Ethics Committees of the Local Health Service as well as the University where the research was conducted. All families recruited to the study provided informed consent to participate, and the research was conducted in accordance with the ethical standards outlined in the Helsinki Declaration.

### 2.2. Development of the Quality of Life in Autism Questionnaire (QoLA)

The QoLA was designed to provide a measure of QoL in parents of children with ASD. The scale was developed for parents of children aged 2–18 years; however specific experimental work in this study was conducted with parents of preschool aged children. This was undertaken for several reasons. First, given increased efforts at early ASD identification in recent years, this group is of particular clinical interest. Second, as this is the first study using the QoLA, the provision of some homogeneity of sample age was considered to be important.

The QoLA was developed following initial consultation and semistructured interviews regarding the impact of caring for a child with ASD upon QoL. These interviews were conducted with consumers and clinicians experienced in working with young people who have ASD and their families. Interviews were transcribed, and a thematic cluster analysis of transcripts was conducted to identify key themes. Item selection and refinement was guided by an expert panel that included several academic clinicians experienced in working with children with ASD from disciplines including psychiatry, psychology, and occupational and speech therapy. Early drafts of the measure were also provided to ASD service provider managers and consumer group representatives for feedback regarding relevance of items and readability. Item selection was ultimately guided by these qualitative data, together with (1) extant models of QoL (e.g., [20]) and (2) review of several widely-used generic QoL measures, as well as (3) consideration of the diagnostic criteria for ASD. An expanded version of the QoL was then piloted with 20 families attending local ASD support services. During the pilot phase, participants were specifically asked whether they felt each item should be included in the measure and were also asked to provide their general thoughts about any areas for improvement. Items rated as not important for inclusion in the scale by >60% of participants during the piloting phase were discussed within the expert panel and all were ultimately omitted in the final QoLA version that was used in the study. Several minor amendments to the wording of questions were also made at that stage based upon the comments of pilot participants so as to improve the readability of the QoLA.

To aid in the clinical utility of the tool and to provide ASD specificity, the QoLA was designed with two subscales (Parts A and B) that are described below. Part A contains some items that are reverse scored. Parents are asked to rate each item based on their experiences over the preceding four weeks.

Part A (QoL subscale) includes 28 items designed to measure parents' overall perception of their quality of life. Items include “I feel happy and content,” “I am satisfied with my social life,” and “I am satisfied with my close relationships”. Each item is measured on a five-point Likert scale ranging from one (not very much) to five (very much). Thus, scores on Part A can range from 28 to 140, with higher scores indicating greater perceived QoL. Items in Part A reflect each of Schalock et al.'s eight domains [20], with a particular weighting on emotional wellbeing, social inclusion, and interpersonal relationships.

Part B (impact of ASD symptoms subscale) was designed to assess parents' perception of how problematic their child's autism-specific difficulties are for them. A list of 20 difficulties that children with ASD can experience was provided (e.g., “socialising with people,” “needing to stick to a routine,” “destructive behaviours including anger and aggression”). Parents are asked to rate how problematic these difficulties have been for them, on a five-point Likert scale from five (not much of a problem for me) to one (very much of a problem for me). Thus, scores on part B can range from 20 to 100, with higher scores denoting fewer problems for parents regarding their child's ASD-related behaviours.

The potential range for the QoLA total score is therefore 48–240. A total score can be computed for overall comparisons; however, Parts A and B were intended to be scored separately and reflect separate subscales within the instrument.

The final version of the QoLA is available free of charge from the study authors and items are outlined in Table 1.

### 2.3. Participants

Two participant groups were recruited: a clinical group comprising parents of children with ASD (ASD group) and a control group comprising parents of children without ASD (control group). Participants were recruited in metropolitan Sydney, Australia, and demographic information for both groups is presented in Table 2. No statistical differences emerged between groups with respect to child age, child sex, parent age, parent sex, or family cultural background. A greater proportion of parents in the control group had a tertiary education.

The ASD group consisted of 23 mothers of children on the waitlist for, or having recently (within the previous month) commenced at, an Autism Specific Early Learning and Care Centre. These children had a mean age of 46.8 months (SD = 9.8, range = 30–71 months), and all had a DSM-IV-TR diagnosis of Autistic Disorder, made by a community-based physician, with the exception of one child who had a diagnosis of Pervasive Developmental Disorder - Not Otherwise Specified. These children would have met DSM-5 criteria for autism spectrum disorder.

A control group was recruited to assist in ascertaining the known-groups validity of the QoLA. This group comprised 16 parents (13 mothers and three fathers) of children attending a childcare centre. None of the children had been diagnosed with ASD or any other developmental problems. The mean age of these children was 43.2 months (SD = 12.1, range = 22–59 months). The absence of an ASD diagnosis among children in the control sample was supported by screening with the Modified Checklist for Autism in Toddlers (M-CHAT, [35]). While several cut-offs are available for the M-CHAT, amongst the most conservative is that advocated by Wong et al, who reported a failing score of six or more of the 23 items using a modified graded scoring system [36]. No children in the control sample failed six or more items on the M-CHAT.

### 2.4. Measures of Quality of Life

In addition to the QoLA, participants in the ASD group also completed the World Health Organisation Quality of Life Questionnaire: WHOQoL-BREF [37]. The WHOQoL-BREF is a 26-item brief version of the original WHOQOL-100 that is designed to assess perceptions of overall quality of life. It generates scores with a range of 0 to 100 on four domains: Physical Health, Psychological Health, Social Relations, and Environment, with higher scores indicating greater perceived quality of life. The WHOQoL-BREF has strong psychometric properties [37] and was the key measure used in the determination of the concurrent validity of QoLA Part A. The WHOQoL-BREF was not completed by the control group due to constraints in the number of measures that could be administered within that setting.

### 2.5. Measures of Severity of Child's Autism Symptoms and Adaptive Functioning

Parents in the ASD group completed two measures to assess the severity of their child's autism symptoms and adaptive functioning. The Social Communication Questionnaire (SCQ: [38]) is a 40-item measure of autism-specific symptoms where scores of 15 or more indicate probable ASD. The SCQ has robust psychometric properties [39, 40]. The Vineland Adaptive Behaviour Scales Second Edition (Parent Form) (VABS-II: [41]) assesses parents' perceptions of their child's everyday adaptive functioning in the domains of Communication (including expressive and receptive language), Daily Living Skills, Socialisation, and Motor Skills. For each domain, including an overall Adaptive Behaviour Composite, a norm-referenced standardised score with a mean of 100 and SD of 15 is calculated. For the maladaptive behaviour index, a *v*-scale score with a mean of 15 and a SD of three is calculated. The VABS-II has well established strong psychometric properties [41]. The SCQ and VABS-II maladaptive behaviour index were used to assist with determining the convergent validity of QoLA Part B. That is, it is reasonable to hypothesise that parent-rated child autism severity (SCQ) and child adaptive functioning (VABS-II) would be related to parents' ratings of how problematic their child's ASD symptoms were for them (QoLA Part B).

### 2.6. Measures of Parental Wellbeing

Participants in the ASD group also completed two additional measures of parental wellbeing. The Depression Anxiety Stress Scales (DASS-21: [42]) is a 21-item self-report measure that assesses negative affect. The measure generates separate scores for the subscales of Depression, Anxiety, and Stress, with higher scores on each subscale indicating greater symptomatology. The DASS-21 has been shown to have excellent psychometric properties [42–44]. The Parenting Sense of Competence Scale (PSOC: [45]) includes 17 items designed to measure parental self-efficacy. Based on the factor structure found in Australian populations [46], the scale generates scores on three subscales: Satisfaction, Efficacy, and Interest, with higher scores indicative of higher levels of parental satisfaction and self-efficacy. The PSOC has been found to have strong psychometric properties [46]. Given the body of research suggesting an association between low QoL and poorer mental health (e.g., [47]), these measures were included to help in the assessment of the convergent validity of the QoLA. The measures were also included to provide additional data regarding the wellbeing of parents whose children have ASD.

### 2.7. Statistical Analysis

Analyses were conducted using SPSS statistical software. Cronbach's alpha values were computed to assess the internal consistency of both Parts A and B of the QoLA. Known-groups validity was evaluated by comparing group QoLA scores in the ASD and control groups. In cases where Levene's test was significant, equal variances were not assumed. Pearson's product-moment correlations were computed to examine the concurrent and convergent validity of the QoLA with the WHOQoL-BREF, parental wellbeing measures, and child functioning measures. Alpha was set at 0.05 for all comparisons, following recommendations by Saville [48] who argues for this per-comparison level rather than a family-wise approach when conducting research in novel areas.

## 3. Results

### 3.1. Descriptive Statistics and Internal Consistency

Descriptive statistics and internal consistency for all measures are summarised in Table 3.

Internal consistency coefficients for the QoLA were *α* = 0.94 for Part A and *α* = 0.92 for Part B in the ASD group, and *α* = 0.96 for Part A and *α* = 0.86 for Part B in the control group. A Cronbach's alpha value of 0.7 to 0.9 is considered to be adequate and greater than 0.9 as excellent [49].

There was some evidence of positive skew in QoLA Part B responses, with parents in the ASD group endorsing the highest response category (very much of a problem for me) 31% of the time on average. This ranged from 65% of parents endorsing the highest response on the item relating to destructive behaviours and aggression to 9% endorsing the highest response for their child needing to stick to a routine.

### 3.2. Construct (Known-Groups) Validity

Independent samples *t*-tests were performed to compare total scores between the clinical and control groups on the QoLA Part A and QoLA Part B. Parents in the ASD group reported significantly lower total scores on the QoLA Part A (mean = 86.96, SD = 21.53) than parents in the control group (mean = 110.00; SD = 17.58), *t*(37) = −3.54, *P* < 0.01). This equates to a Cohen's *d* effect size of 1.17, which is large. Parents in the ASD group also reported significantly lower total scores on the QoLA Part B (mean = 68.52, SD = 17.56) than parents in the control group (mean = 94.31, SD = 6.14), *t*(29.09) = −6.50, *P* < 0.001, equivalent to Cohen's *d* of 1.96, which is again large.

In terms of the QoLA total score, the ASD group reported significantly lower scores (mean = 155.48, SD = 26.86) than parents in the control group (mean = 204.31, SD = 19.64), *t*(36.87) = −6.59, *P* < 0.001, equivalent to Cohen's *d* of 2.08.

### 3.3. Concurrent and Convergent Validity

Scores on the QoLA Part A were positively correlated with scores on all four subscales of the WHOQOL-BREF (*r* = 0.74 to 0.91, all *P*s < 0.01) suggesting strong concurrent validity. Furthermore, QoLA Part A was associated negatively with maternal depression (*r* = −0.49, *P* < 0.05) and stress (*r* = −0.57, *P* < 0.01) and positively with maternal satisfaction (*r* = 0.74, *P* < 0.01). Moderate sized correlations (>0.30) in the expected direction were also observed with maternal anxiety and maternal efficacy.

Scores on the QoLA Part B approached significance in correlation with the SCQ (*r* = −0.37, *P* = 0.086) and a moderate and identical sized correlation of *r* = −0.37 was also observed with the VABS-II maladaptive behaviour index. This provides some support for the convergent validity of the QoLA Part B. QoLA Part B scores were not significantly correlated with scores on the QoLA Part A, nor any of the subscales of the WHOQOL-BREF, DASS-21, or PSOC. See Table 4.

### 3.4. Comparison with Normative Data

To further explore the psychosocial functioning of parents in the ASD group, mean scores for the WHOQOL-BREF, DASS-21, and PSOC in the clinical group sample were compared to Australian population norms [37, 43, 46] using Welch *t* tests. Mothers in the ASD group had significantly lower scores on the Physical (*t*(24) = 4.43, *P* < 0.0001), Psychological (*t*(24) = 5.06, *P* < 0.0001), Social Relations (*t*(23) = 3.64, *P* < 0.01), and Environmental (*t*(23) = 4.43, *P* < 0.001) domains of the WHOQOL-BREF than the Australian population norms [37]. Mothers in the current sample also reported significantly higher levels of Depression (*t*(43) = 3.07, *P* < 0.01), Anxiety (*t*(29) = 3.08, *P* < 0.01), and Stress (*t*(44) = 3.43, *P* < 0.01) on the DASS-21 than nonclinical volunteers in the study by Antony et al. [43]. There were no significant differences between mothers' scores on Satisfaction (*t*(23) = 1.31, *P* = 0.20) or Interest (*t*(22) = 1.85, *P* = 0.08) in the ASD group and those reported by Rogers and Matthews [46], but mothers in the current sample were found to have significantly higher scores on Efficacy (*t*(23) = 5.69, *P* < 0.0001).

### 3.5. Association between ASD Symptoms and Parental Wellbeing

There were no significant correlations between scores on the QoLA Part B or SCQ and scores on any measures of parental wellbeing (QoLA, WHOQoL-BREF, DASS-21, or PSOC). See Table 4.

## 4. Discussion

To date the assessment of QoL in parents of children with ASD and the broader incorporation of this construct into clinical settings have been complicated by the lack of an English speaking ASD-specific measure. The results of the present study suggest that the QoLA may provide an initial step toward remedying this situation.

The primary aim of this paper was to describe the conceptualisation and development of the QoLA. Given the relatively small sample size involved in the study, the psychometric properties reported should be considered preliminary. Nonetheless, QoLA Part A shows excellent internal consistency as well as strong concurrent validity with the WHOQOL-BREF and good convergent validity with measures of maternal depression, stress, and parenting satisfaction. QoLA Part B also showed excellent internal consistency, approached significance in its convergent association with a measure of ASD symptomatology, the Social Communication Questionnaire, and showed a moderate correlation with the VABS-II maladaptive behaviour index. Both QoLA Part A and B and the QoLA total score showed strong known-groups validity between an ASD and nonclinical cohort. Together, these results provide promising initial data on the reliability and validity of the QoLA.

Given the QoLA Part B is relatively unique in asking parents of children with ASD how much of a problem a range of ASD-specific behaviours are for the parent, locating a measure that was directly comparable for the establishment of validity was problematic. More specifically, while the SCQ enquires about ASD symptoms and the VABS-II maladaptive behaviour scale about challenging behaviours, neither specifically enquires after the parent's perceptions of how much of an issue these behaviours are to the parent. In this regard, the medium sized associations observed (*r* = 0.37) between QoLA Part B and both the SCQ and VABS-II maladaptive behaviour index do not necessarily challenge the validity of this part of the scale. Moreover, given QoLA Part B was constructed in part with reference to DSM criteria, the face validity of items and relevance to clinical practice is high. The authors would contend that QoLA Part B also offers clinical utility in identifying those families for targeted additional support where the child's ASD symptoms are perceived as highly problematic to the parent.

Results of this study provided support to the extensive existing literature highlighting the psychosocial difficulties faced by parents of children with ASD [50] and help to extend these findings to parents of preschool aged children. Specifically, when compared to available nonclinical normative data, participants in this study showed lower QoL scores across the WHOQoL-BREF domains of Physical, Psychological, Social Relations, and Environmental QoL. In addition, mothers of children with ASD in the present sample were found to have higher depression, anxiety, and stress scores than those reported in nonclinical normative data. Interestingly, mothers of children with ASD in the current sample were found to have higher efficacy scores than those reported in Australian nonclinical normative data. The Efficacy subscale of the PSOC includes items assessing parents' perceptions of their parenting capability. It is conceivable that, due to the unique challenges associated with parenting a child with ASD and the availability of specialist assistance to parents in our cohort, some parents may develop greater confidence in their ability as parents to manage difficult situations. Replication of this finding would be necessary before firm conclusions could be drawn, however.

While the primary aim of the present study was to develop the QoLA, one unexpected set of findings generated in the conduct of the study related to the apparent lack of relationship between ASD symptomatology and measures of parental QoL and parental wellbeing. That is, there were no significant correlations between either the QoLA Part B or SCQ (i.e., measures, broadly speaking, of ASD symptomatology) and the QoLA Part A, WHOQOL-BREF, or DASS-21 subscales (i.e., measures of QoL and parental wellbeing). This may suggest that the children's ASD symptoms themselves may not necessarily be the primary factor affecting QoL and other indices of wellbeing among parents and caregivers. This finding is consistent with studies that have reported that factors outside of ASD symptom severity per se may contribute to increased psychological distress among parents of children. These factors include low levels of perceived social support [51–53] and psychological characteristics of the parent such as low perceived self-efficacy [54], external locus of control, and emotion-focused coping styles [23, 53]. This finding is also consistent with literature demonstrating that QoL among adults with ASD is not predicted by the severity of their disability but rather by the availability of social support [55].

Scores on the Environmental subscale of the WHOQOL-BREF in the present study were found to be negatively correlated with maternal depression, anxiety, and stress and positively correlated with all other domains on the WHOQOL-BREF and the QoLA Part A. This observation is consistent with research demonstrating that parents with greater perceived access to social support have lower levels of psychological distress [51–53]. Bromley et al. [51] observed that mothers were more likely to report lower levels of support if they were a lone parent, were living in poor housing, or were the mother of a boy with ASD.

It also appears that the age of the child may be an important mediator in determining parental wellbeing, with one study observing that there was greater family stress when the child with autism was an older adolescent or adult (aged 15–21.9 years) as compared to a younger adolescent (aged 10–14.9 years) [11]. In this regard, by four years of age—the approximate average age of ASD probands in the present study—a cumulative psychosocial burden may not yet have developed for some parents.

Nonetheless, numerous studies have shown that parents' level of psychological distress is associated with the severity of their child's ASD symptoms and challenging behaviours [51, 52, 56–59]. In particular, it has been shown that disruptive behaviours, fixed schedules, and difficulties for families participating in activities outside home are the main reasons for family burden [60]. Parents of children with ASD have also been found to be at increased risk of experiencing both physical and psychological distress [14, 61, 62] with the demands of caring for a child with special needs such as ASD resulting in parents having less time to meet their own needs as well as limited opportunities for participation in employment and leisure activities.

Given children in the present sample had a relatively high level of ASD symptomatology and were from a group that warranted receipt of early intensive behavioural intervention, it is also possible that a modest ceiling effect was in operation in the data for ASD symptomatology, potentially limiting variability, and experimental power for detecting effects of ASD symptoms upon wellbeing.

Limitations of the current study need to be taken into account when interpreting findings. The preliminary nature of the psychometric data given the small sample size has been noted above. Further, while the QoLA is thought to be useful for parents of children aged up to 18 years, given the present study focused upon preschool aged children, it is not yet clear whether results will generalise to cohorts with older children. Similarly, while the authors would contend that the QoLA is applicable to fathers, further research with fathers is required before this can be substantiated as the present clinical sample involved only mothers. In addition, only parent self-report measures were utilised to ascertain the child's level of autism severity and adaptive functioning, rather than a standardised observational measure of ASD diagnosis and severity. Finally, we did not directly ascertain challenging behaviours in the child or the availability of psychosocial support for the parents, factors known to impact on parental wellbeing. Future research will need to address these issues, in addition to examining the QoLA in older children as well as determining the utility of a parallel self-report version of the measure that has been designed for use amongst adolescents and adults with ASD. Further work examining the psychometric properties of the scale will also be crucial, particularly its test retest reliability and factor structure, as will additional experimental work examining the levels of agreement between parents with respect to their perceptions of their quality of life.

## 5. Conclusions

The present study reports on the conceptualisation and development of the QoLA. To the authors' knowledge, this is the first English-speaking measure of autism-specific QoL for parents of children with ASD that has been developed. The preliminary psychometric properties of the scale are encouraging with each of the two subscales of the QoLA showing strong internal consistency and good convergent validity. Moreover, the QoLA subscales and total score showed excellent discrimination between an ASD and control sample. Future work expanding on the psychometric properties of the QoLA would appear to be indicated, as is research more generally that examines QoL in families of young people affected by ASD.

## Figures and Tables

**Table 1:** QoLA items.

	QoLA—Part A (QoL subscale)	QoLA—Part B (impact of ASD symptoms subscale)
(1)	I am satisfied with my life	Socialising with people
(2)	I feel stressed*	Having friends
(3)	I feel happy and content	Understanding others' feelings
(4)	I feel depressed or anxious*	Holding a conversation
(5)	I feel good about myself as a person	Communicating needs
(6)	I am satisfied with my close relationships	Taking a literal meaning of comments
(7)	People are there for me when I need them	Saying things that are socially embarrassing
(8)	I am satisfied with my social life	Needing to stick to a routine
(9)	I am satisfied with my family life	Being overly interested in a particular topic
(10)	I am satisfied with my financial situation	Getting anxious in a specific situation or during changes
(11)	I am satisfied with where I live	Sensitivity to certain sensations
(12)	I have enough money to meet my needs	Understanding the rules of social interaction
(13)	I am satisfied with my achievements	Managing emotional responses
(14)	I am satisfied with my general health	Needing to do things a certain way
(15)	I have a healthy lifestyle	Destructive behaviours including anger and aggression
(16)	I am satisfied with my leisure activities	Showing inappropriate emotional reactions
(17)	Health problems stop me doing things that I want to*	Unusual repetitive behaviours or body movements
(18)	I feel in control of my life	Engaging in reckless or tactless behaviours
(19)	I set and achieve goals in my life	Doing daily living tasks independently
(20)	I can make a plan of action and follow it	Responding when approached socially
(21)	I make my own decisions	
(22)	I feel guilty*	
(23)	I am part of a community	
(24)	I can get the support that I need from the community	
(25)	I am able to get to where I need to	
(26)	I feel safe in my everyday life	
(27)	I feel respected in my everyday life	
(28)	I am satisfied with the availability of health services	

*Item is reverse scored.

**Table 2:** Demographic information for the ASD and control groups.

	ASD group	Control (non-ASD) group
*n*	23	16

Mean child age in months (SD)	49.39 (16.75)	43.19 (12.13)

Number with ASD	23	0

Child gender Male : Female	17 : 6	10 : 6

Mean parent age in years (SD)	35.10 (4.50)	37.88 (3.40)

Parent gender Male : Female	0 : 23	3 : 13

Parent's education background	47% tertiary	100% tertiary
	48% secondary	
	5% no formal education	

Parent's ethnic background	47% Australian or New Zealander	63% Australian or New Zealander
	32% South-East Asian	25% South-East Asian
	16% European	6% European
	5% Middle Eastern	6% Southern Asian

**Table 3:** Descriptive statistics and internal consistency coefficients for all measures used.

Variable	Mean	Range	SD	Cronbach's *α*
QoLA Part A: ASD group	86.96	46–138	21.53	0.94
QoLA Part A: control group	110.00	65–138	17.58	0.96
QoLA Part B: ASD group	68.52	30–99	17.56	0.92
QoLA Part B: control group	94.31	78–100	6.14	0.86
WHOQOL-BREF: Physical^a^	59.32	28.57–89.29	17.45	0.85
WHOQOL-BREF: Psychological^a^	55.98	33.33–91.67	15.38	0.75
WHOQOL-BREF: Social relations^a^	55.07	16.67–100	22.15	0.72
WHOQOL-BREF: Environmental^a^	58.70	28–97	17.12	0.84
DASS-21: Depression^a^	4.91	0–13	3.57	0.86
DASS-21: Anxiety^a^	3.30	0–10	3.01	0.75
DASS-21: Stress^a^	6.74	0–14	3.70	0.81
PSOC: Satisfaction^a^	3.16	1–5.4	1.02	0.81
PSOC: Efficacy^a^	4.35	2.14–6	0.95	0.89
PSOC: Interest^a^	4.59	2–6	1.35	0.78

^a^Measure administered to ASD group only.

**Table 4:** Correlations between all measures in the ASD sample.

Measure		2	3	4	5	6	7	8	9	10	11	12	13	14	15	16	17	18	19
(1)	QoLA Part A	−.07	.74**	.82**	.78**	.91**	−.49*	−.40	−.57**	.74**	.38	.26	−.12	.12	.09	.08	.10	−.02	.15
(2)	QoLA Part B	—	−.03	−.16	−.18	−.22	.19	.17	.14	.26	−.05	.22	−.37	.29	.25	−.18	.05	−.37	.23
(3)	WHO Physical		—	.63**	.69**	.75**	−.26	−.26	−.46*	.62**	.16	−.00	−.25	−.13	−.22	.23	.06	−.25	.08
(4)	WHO Psychological			—	.64**	.87**	−.45*	−.28	−.38	.52*	.26	.23	−.15	.31	.18	.33	.18	.20	.34
(5)	WHO Social				—	.72**	−.36	−.33	−.50*	.67**	.32	.21	.21	.02	−.11	−.04	.17	.07	−.02
(6)	WHO Environment					—	−.56**	−.44*	−.59**	.69**	.29	.23	−.22	.00	.14	.08	−.03	.06	.05
(7)	DASS-21: Depression						—	.66**	.81**	−.43*	−.45*	−.18	−.18	−.10	−.20	−.24	−.03	−.17	−.16
(8)	DASS-21: Anxiety							—	.73**	−.47*	−.27	−.17	−.00	.14	−.10	−.01	.15	.22	.09
(9)	DASS-21: Stress								—	−.58**	−.56**	.04	.09	.17	−.02	−.06	.13	.13	.10
(10)	PSOC: Satisfaction									—	.23	.30	−.24	.05	.12	−.07	.16	−.18	.13
(11)	PSOC: Efficacy										—	.31	.04	.25	.06	−.01	.28	.01	.19
(12)	PSOC: Interest											—	.10	.49*	.05	−.26	.26	.25	.22
(13)	SCQ Total												—	−.00	−.55**	−.44*	−.04	.29	−.33
(14)	VABS Communication													—	.39	.18	.50*	.45*	.74**
(15)	VABS Socialisation														—	.45*	.48*	−.05	.75**
(16)	VABS Daily Living															—	.42	.28	.63**
(17)	VABS Motor Skills																—	.30	.82**
(18)	VABS Maladaptive																	—	.35
(19)	VABS Adaptive Behaviour Composite																		—

Note: **P < .01; *P < .05. QoLA: Quality of Life in Autism Questionnaire; WHO: World Health Organisation Quality of Life Questionnaire: brief version; DASS-21: Depression, Anxiety, Stress Scale 21; PSOC: Parent Sense of Competence Questionnaire; SCQ: Social Communication Questionnaire; VABS: Vineland Adaptive Behaviour Scales 2nd Edition.
